# Data analysis of “krokodil” samples obtained by street-like synthesis

**DOI:** 10.1016/j.dib.2015.11.046

**Published:** 2015-11-28

**Authors:** João Filipe Neves, Emanuele Amorim Alves, José Xavier Soares, Sara Manuela Cravo, Artur M.S. Silva, Annibal Duarte Pereira Netto, Félix Carvalho, Ricardo Jorge Dinis-Oliveira, Carlos Manuel Afonso

**Affiliations:** a Department of Chemical Sciences, Laboratory of Organic and Pharmaceutical Chemistry, Faculty of Pharmacy, University of Porto, Porto, Portugal; b UCIBIO-REQUIMTE, Laboratory of Toxicology, Department of Biological Sciences, Faculty of Pharmacy, University of Porto, Porto, Portugal; c Department of Legal Medicine and Forensic Sciences, Faculty of Medicine, University of Porto, Porto, Portugal; d EPSJV – Polytechnic School of Health Joaquim Venâncio, Oswaldo Cruz Foundation, Rio de Janeiro, Brazil; e LAQV-REQUIMTE, Department of Chemical Sciences, Laboratory of Applied Chemistry, Faculty of Pharmacy, University of Porto, Porto, Portugal; f Department of Chemistry and QOPNA, University of Aveiro, Campus Universitário de Santiago, Aveiro, Portugal; g Department of Analytical Chemistry, Chemistry Institute, Fluminense Federal University, Niterói, Brazil; h IINFACTS-Institute of Research and Advanced Training in Health Sciences and Technologies, Department of Sciences, University Institute of Health Sciences (IUCS), CESPU, CRL, Gandra, Portugal; i Interdisciplinary Center of Marine and Environmental Investigation (CIIMAR/CIMAR), Porto, Portugal

**Keywords:** “Krokodil”, Street like synthesis, TLC profile, UV/Vis, ^1^H NMR, FTIR

## Abstract

The data described in this work is related to be the subject of an article in the Forensic Science International, titled: “The harmful chemistry behind “krokodil”: street-like synthesis and product analysis” (http://dx.doi.org/10.1016/j.forsciint.2015.07.042) [1]. The data presented here provides additional description of the chemical profile of “krokodil”. Physicochemical and organoleptic characteristics, TLC profile, UV/Vis, ^1^H NMR and FTIR spectrum are presented. These data validate the proposed synthetic procedure and pathway and give further information about the contaminants present in “krokodil”.

**Specifications Table**

**Table:** 

Subject area	*Chemistry*
More specific subject area	*Chemical profile data*
Type of data	*Figures*
How data was acquired	*UV analysis (Varian CARY 100 spectrophotometer from a range of 200 nm to 800 nm. Software: Cary Win UV, v. 3.0), FTIR analysis (Nicolet iS10 from Thermo Scientific. Smart OMNI-Transmisson accessory. Software OMNIC 8.3) and ^1^H NMR analysis (Bruker DRX-300 spectrometer operating at 300.13 MHz for*^*1*^*H)*
Data format	*Analyzed data*
Experimental factors	*Additional chemical profile data from “krokodil” samples*
Experimental features	*Crude “krokodil” obtained using street-like synthesis and its extract after alkalization and organic extraction using ethyl acetate as solvent were analyzed*
Data source location	*Porto, Portugal*
Data accessibility	*Data is provided in this article*

**Value of the data**

• Detailed description of organoleptic properties and pH range of “krokodil” as well as the disclosure of UV/Vis and ^1^H NMR spectra provide additional data to the establishment of the chemical profile of “krokodil”.
• The description of the chemical profile of “krokodil” will eventually aid the competent authorities in dealing with this drug, in terms of identification and characterization.
• Further insight regarding the complex nature of “Krokodil” was revealed by TLC analysis and FTIR spectrum.

## 1 Data

Data presented here describes the additional chemical analysis of the “krokodil” samples obtained using the street-like synthesis. Physical and organoleptic characters, UV/Vis and ^1^H NMR spectra were described on a “krokodil” sample freshly prepared (crude “krokodil”). Organic extract of “krokodil” (extracted “krokodil”) was obtained after alkalization of the crude product and extraction using ethyl acetate. This organic extracts were analyzed by TLC, FTIR and ^1^H NMR techniques.

## 2 Experimental design, materials and methods

The synthesis was carried out as described previously [1]. “Krokodil extract” samples were obtained by the treatment of 4 mL of crude “krokodil” with NaOH 20% (m/v) until alkalization, followed by extraction with ethyl acetate. The organic phases were gathered, dried over anhydrous sodium sulfate, filtered and concentered until dryness.

All pH measurements were made with a Model pH-meter GLP 22 (Crison, Allela, Spain).

UV/Vis spectra of water-diluted solutions of crude “krokodil” were recorded on a Varian CARY 100 spectrophotometer from a range of 200 nm to 800 nm (software: Cary Win UV, v. 3.0). ^1^H NMR spectrum was recorded on a Bruker DRX-300 spectrometer (operating at 300 MHz for ^1^H) using D_2_O (Deutero GmbH) as solvent.

TLC experiments were carried out on pre-coated plates (silica gel, 60 F254 Merck) with 0.2 mm of thickness. Elution took place at a CAMAG Horizontal Developing chamber and five mobile phases were tested. Chromatograms visualization was conducted under UV light at 254 and 365 nm.

FTIR spectrum was obtained in a FTIR spectrometer Nicolet iS10 from Thermo Scientific, using KBr disks. Spectra analysis was performed with Smart OMNI-Transmission accessory (Software OMNIC 8.3).

Crude “krokodil” appeared as a yellow to light brown solution due to the presence of iodine [2] and with a very characteristic acidic smell. The final product did not reveal any signal of iodine crystals and red phosphorus sediments were successfully removed by filtration. The pH of crude “krokodil” samples was 1.15±0.30. The low pH value is in accordance with the literature [1], [2]. However, this is the first time that pH value was reported with analytical precision.

**Fig. 1:**
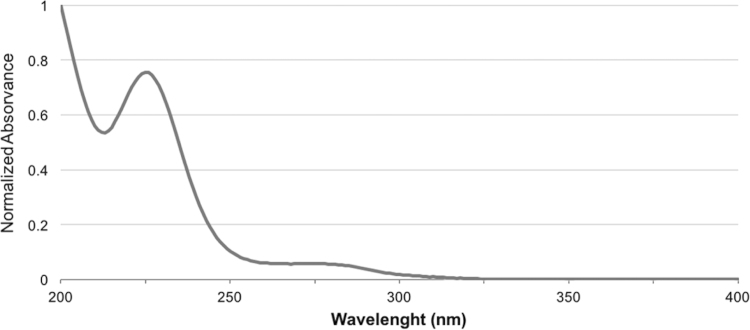
UV/Vis spectrum of crude “krokodil”.

UV/Vis spectrum of crude “krokodil” was performed to evaluate the presence and extension of chromophores (Fig. 1). Two main absorption bands were observed in the spectrum, one in the range of 215–250 nm (*λ*_max_ at 225 nm) and other in the range of 250–300 nm (*λ*_max_ at 276 nm). Absorptions in the 215–250 nm range are associated with presence of organic substances with unsaturated bonds and few conjugated systems (π→π* transitions). The absorptions in the 250–300 nm range are associated with organic substances with stronger chromophores and also with auxochromes (n→π* transitions) or conjugated systems [3]. It is noteworthy, that the band in the 250–300 nm range is compatible with the *λ*_max_ of absorption of some morphinans (*λ*_max_ 284 nm for desomorphine and *λ*_max_ 285 nm for codeine) [4], [5].

**Fig. 2:**
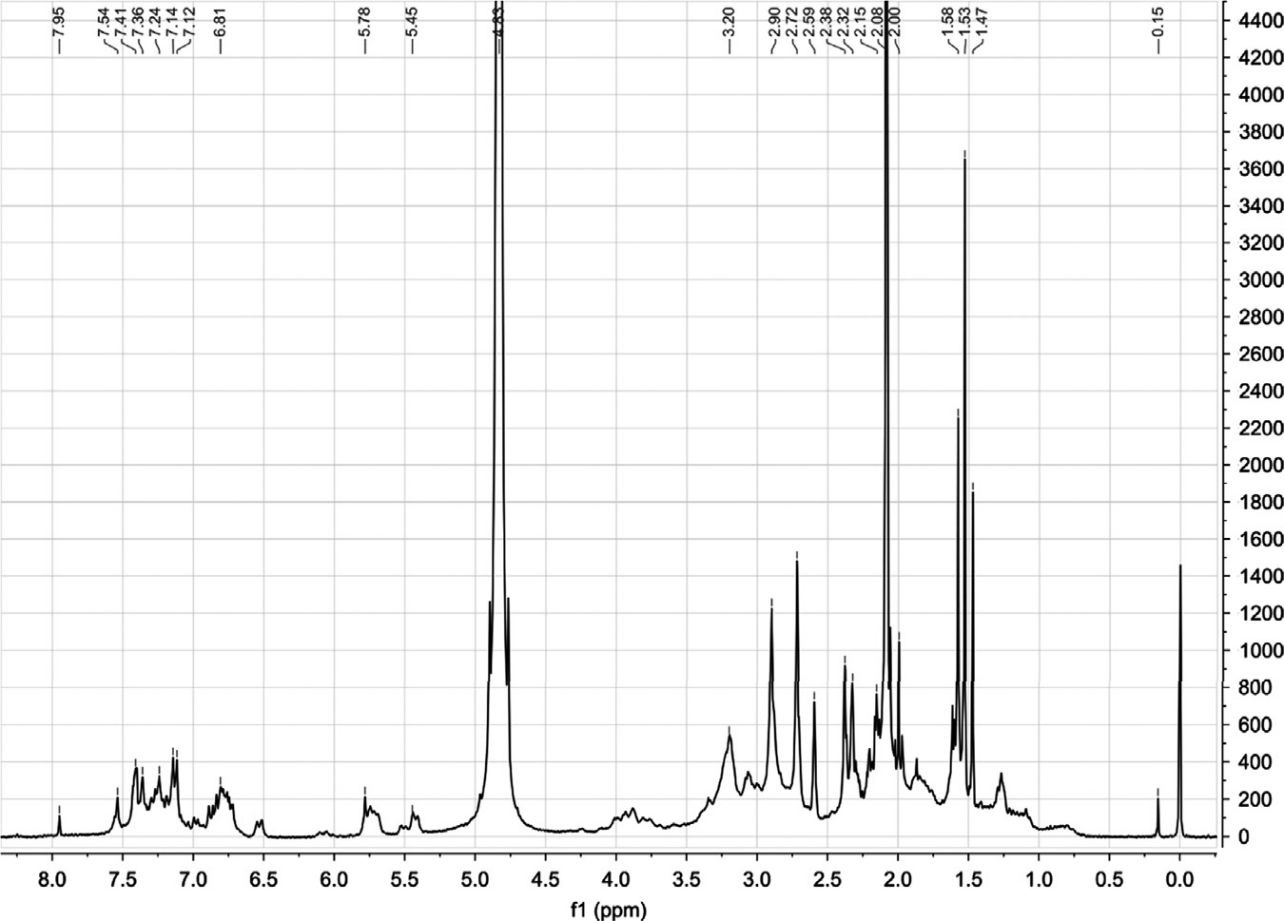
^1^H NMR spectrum of crude “krokodil”.

^1^H NMR spectrum of crude “krokodil” was also recorded (Fig. 2). The spectrum exhibits a lower frequency value signal (*δ*=0.15 ppm) which may be due to the presence/contamination with some raw materials used in the synthesis, like silicone grease (polydimethylsiloxane) or a similar compound [6]. The presence of several signals in the range of 0.5–2 ppm, was also observed, suggesting the presence of aliphatic protons and several signals in the range of 2–3.5 ppm, suggesting the presence of heteroatoms adjacent to carbons. As “krokodil” is an aqueous solution, D_2_O was used as solvent for NMR analysis. The residual H_2_O signal (D_2_O was not 100% deuterated), a broad signal at 4.83 ppm, can overlap proton signals due to alcohols, phenols, amides or amines [7]. The absorptions in the range of 5–6 ppm are compatible with vinylic protons. A substantial number of different aromatic protons are also present once there are several signals in the range of 6.5–8 ppm.

In “krokodil” manufacture, organic and inorganic reactants are involved and it is agreed that organic and inorganic compounds are produced. Considering the organic compounds present in “Krokodil”, morphinans are very important, since they are the responsible for the psychoactive effects of this drug. In order to study these organic components, crude “krokodil” was basified with sodium hydroxide and extracted with ethyl acetate yielding an organic extract designated as “krokodil extract”. The treatment of crude “krokodil” with a strong base assured the presence of morphinans as free bases, which were subsequently extracted with ethyl acetate, an organic solvent of intermediate polarity [8]. However, these procedures are not specific for morphinan molecules, since all organic compounds, with the exception of acidic substances, are also extracted.

**Fig. 3:**
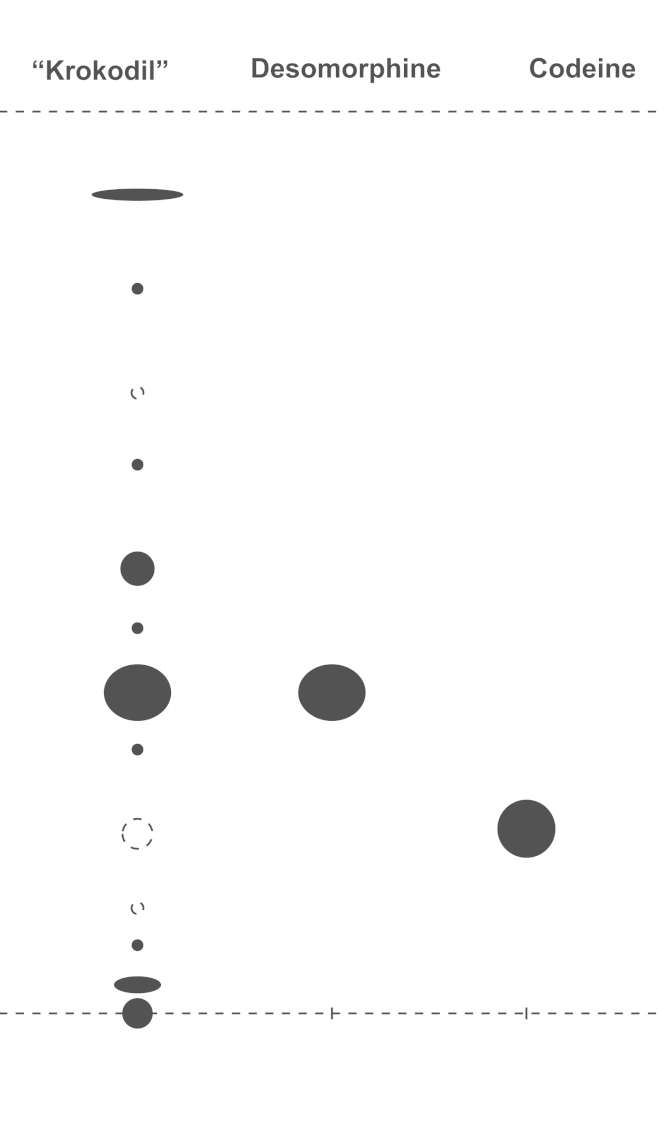
Thin-layer chromatographic profile of extracted “krokodil” samples. Mobile phase: hexane/ethyl acetate/diethylamine (6:4:0.5).

Taking in account the fact that “krokodil” contains a mixture of morphinans, several mobile phases described in the literature for thin-layer chromatography (TLC) analysis of morphinans were tested [9], [10]. For the TLC analysis a mixture of hexane/ethyl acetate/diethylamine was selected as mobile phase (6:4:0.5). The chromatographic profile of “krokodil extract” with this mobile phase is shown in Fig. 3.

**Fig. 4:**
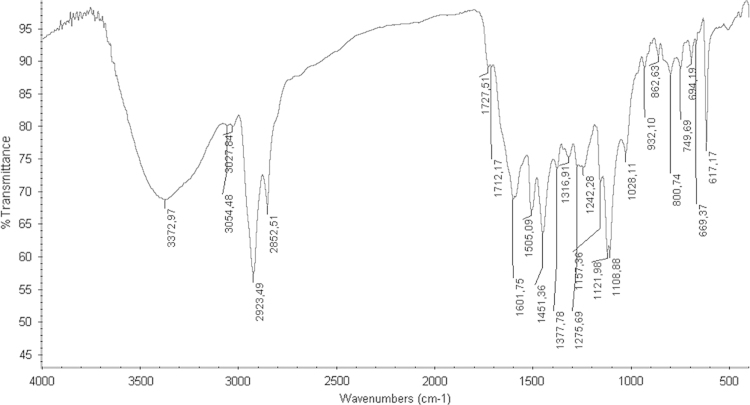
– FTIR Spectrum of “krokodil” in KBr microplate.

The FTIR spectrum of “krokodil” showed an absorption compatible with a phenolic hydroxyl (3382 cm^−1^, m, PhO–H stretch), a group present in acetaminophen, desomorphine and morphinan-4,5-epoxy-3-ol (Fig. 4). The presence of vinylic carbons, which was already recognized in ^1^H NMR spectrum, is also suggested (3028 cm^−1^, m, =C–H stretch). In accordance with the ^1^H NMR spectrum, FTIR spectrum also shows several bands compatible with the aromatic C=C stretching (1559 cm^−1^, m; 1542 cm^−1^, m; 1509 cm^−1^, m; 1456 cm^−1^, m).

## Conflict of interest statement

Authors declare no conflict of interest.
